# Exploring predictive factors of physiological, biochemical indicators, and lifestyle for macrovascular complications in type 2 diabetes: a synthesis of machine learning models

**DOI:** 10.3389/fendo.2026.1696240

**Published:** 2026-02-17

**Authors:** Bo Shang, Yaoqin Lu, Chengjing Wei, Yunlong Li, Qingyue Yang, Yukai Li, Sheng Jiang, Yinxia Su

**Affiliations:** 1 College of Medical Engineering Technology, Xinjiang Medical University, Urumqi, Xinjiang, China; 2 Xinjiang Uygur Autonomous Region Center for Disease Control and Prevention, Urumqi, Xinjiang, China; 3 School of Public Health, Xinjiang Medical University, Urumqi, Xinjiang, China; 4 Administrative Office, Yili Normal University, Yining, Xinjiang, China; 5 The Department of Endocrinology, The First Affiliated Hospital of Xinjiang Medical University, Urumqi, Xinjiang, China; 6 State Key Laboratory of Pathogenesis, Prevention and Treatment of High Incidence Diseases in Central Asia, Urumqi, Xinjiang, China

**Keywords:** machine learning, macrovascular complications, predictive modeling, type 2 diabetes mellitus, XGBoost

## Abstract

**Background:**

Traditional risk models for macrovascular complications in type 2 diabetes (T2DM) rely on physiological and biochemical indicators, which may lack long-term follow-up data and thus potentially overlook key variables.

**Methods:**

A retrospective cohort study was conducted on 4,186 T2DM patients from the Diabetes Health Management Platform in Hotan, Xinjiang, covering the period from 2015 to 2023. Eight machine learning (ML) algorithms were used, with an 8:2 random split into training (n=3,348) and validation (n=838) sets. Performance was evaluated using the area under the receiver operating characteristic curve (AUC), and feature contributions were analyzed using SHAP values. The clinical applicability was verified through decision curve analysis.

**Results:**

The T2DM with macrovascular complications group had significantly higher waist circumference, oropharyngeal abnormalities, and absent lung crackles (*P* < 0.05). The T2DM with macrovascular complications group also had significantly higher BMI, body temperature, and ALT (*P* < 0.05), but lower fasting blood glucose, with borderline abnormalities in blood urea and AST. The T2DM with macrovascular complications group had higher smoking, alcohol consumption, and exercise frequency (*P* < 0.05), but a reverse trend in self-reported “poor” health status (*P* < 0.05). Among all machine learning models (training AUC 0.68-0.85), XGBoost performed best (training AUC = 0.830, validation AUC = 0.850), with superior clinical net benefit compared to traditional strategies. SHAP analysis revealed that BMI (contribution +0.1116), body temperature (+0.0923), and LDL-C (+0.0821) were key predictive factors, with elevated body temperature potentially indicating subclinical inflammation activation.

**Conclusions:**

Among patients with vascular complications, the disconnect between health behavior risks and subjective health perception is more pronounced. Elevated body temperature, high blood pressure, triglycerides, and fasting glucose indicate inflammation, increasing cardiovascular risk; moderate regular exercise provides protection.

## Introduction

Type 2 Diabetes Mellitus (T2DM) is one of the most severe and rapidly growing public health problems in modern times, with approximately 500 million people affected globally as of 2024, accounting for about 10% of the global population (1, 2). Among the challenges faced by T2DM patients, complications, particularly macrovascular complications, are a leading cause of mortality, accounting for 70%-80% of deaths, including coronary artery disease, cerebrovascular accidents (stroke), and peripheral artery disease. These significantly increase mortality and morbidity rates (3, 4). In addition to macrovascular complications, microvascular complications, including diabetic retinopathy, neuropathy, and nephropathy, also contribute to the overall impact of T2DM. The prevalence of this disease shows significant regional heterogeneity, with higher rates in areas with advanced urbanization and industrialization, and a rapidly increasing incidence in less developed regions with relatively scarce resources. This cross-socioeconomic gradient pattern reflects the global spread of the disease (5–7). Furthermore, global population aging is expected to exacerbate the disease and economic burden of T2DM and its related complications, not only increasing healthcare costs and placing enormous demand on global healthcare systems but also imposing significant psychological burdens on patients and their families (8–10). Over the past few decades, researchers have delved into the impact of diabetes and vascular diseases on health using various biomedical technologies. Blood glucose control is crucial for preventing diabetic complications. Several clinical studies have shown that strict blood glucose control can significantly reduce the incidence of cardiovascular diseases (11, 12). Additionally, the development of anti-diabetic drugs has been progressing steadily, with many new medications, such as SGLT-2 inhibitors and GLP-1 receptor agonists, effectively controlling blood glucose levels and showing protective effects against macrovascular diseases (13, 14).Simultaneously, controlling blood lipids and blood pressure is also critical in preventing diabetes and macrovascular diseases (15). Active control of blood lipids and blood pressure not only reduces the risk of cardiovascular events in diabetic patients but also improves their overall prognosis (16). Exercise, dietary interventions, and lifestyle improvements have also been shown to play a positive role in the prevention and treatment of diabetes (17). Despite the current measures and treatments for T2DM, the prevention and treatment of its macrovascular complications still face many challenges.

T2DM macrovascular complications are primarily triggered by long-term hyperglycemia and metabolic abnormalities, leading to changes in the structure and function of blood vessel walls, which in turn exacerbate arteriosclerosis, restrict blood flow, and increase the risk of cardiovascular events (18). The mechanisms of macrovascular complications are complex and involve interactions of multiple factors (19).Traditional risk prediction models for T2DM complications mainly rely on clinical and demographic factors such as age, sex, blood pressure, and blood lipids (20).These models often fail to capture the multifactorial and dynamic nature of T2DM and its complications, leading to limited predictive accuracy, which may not fully support personalized decision-making in clinical practice (21, 22). The emergence of machine learning (ML) technology provides a promising alternative to traditional methods, integrating and analyzing large and complex datasets to identify patterns and relationships that may have been overlooked. ML algorithms have shown excellent performance in various medical applications, including oncology, cardiology, and nephrology, and have been used to predict disease progression, optimize treatment strategies, and improve patient outcomes (23–25).ML models exhibit tremendous potential in integrating multiple types of data, including clinical, biochemical, genetic, and lifestyle factors.

Moreover, primary care settings often lack adequate screening capacity and patient engagement, and there is a notable absence of a universally validated prediction model. Although previous studies have employed machine learning algorithms to predict macrovascular complications in T2DM, few have thoroughly explored the integration of multi-factorial and multidimensional data. Health check-ups and follow-up data play a crucial role in the study of macrovascular complications in diabetes (26–28). Follow-up data provide valuable insights into the long-term progression of the disease, help assess the effectiveness of treatment strategies, and uncover the mechanisms underlying macrovascular complications. Currently, research on T2DM macrovascular complications is mainly focused on experimental and clinical studies (29). Existing research often concentrates on individual environmental or genetic factors, with limited discussion on the combined effects of these factors. Additionally, studies incorporating large-scale lifestyle follow-up data and environmental factors are relatively scarce (30). Therefore, integrating lifestyle follow-up data with genetic and environmental factors to build a clinical prediction model that incorporates clinical physiological and biochemical indicators can enhance the detection rate of complications in T2DM patients.

This study aims to compare eight robust machine learning-based prediction models for identifying risk factors associated with macrovascular complications in patients with type 2 diabetes mellitus. We systematically evaluate 52 clinical, biochemical, and lifestyle-related variables and compare the performance of multiple machine learning algorithms to construct an accurate and generalizable predictive model (31, 32). The primary objective is to provide clinicians with a non-invasive and cost-effective tool for early identification of high-risk individuals, enabling timely interventions to prevent or delay the onset of macrovascular complications (33). Furthermore, the findings may inform public health strategies to reduce the burden of diabetes-related macrovascular diseases, thereby contributing to more sustainable healthcare systems and improved quality of life for patients (34, 35).

## Methods

### Study population

The study population for this research consists of residents with Type 2 Diabetes Mellitus (T2DM) from the Hotan region of Xinjiang Uygur Autonomous Region. By coordinating with primary healthcare institutions, we obtained standardized diabetes management follow-up data collected by local healthcare service providers. The study covers eight counties and cities under the jurisdiction of Hotan, including Pishan County, Hetian County, Hetian City, Moyu County, Cele County, Minfeng County, Luopu County, and Yutian County. In addition, this study simultaneously collected health examination data from county-level people’s hospitals in these eight counties and cities during the 2015–2023 period, specifically from Pishan County People’s Hospital, Hetian County People’s Hospital, Hetian City People’s Hospital, Moyu County People’s Hospital, Cele County People’s Hospital, Minfeng County People’s Hospital, Luopu County People’s Hospital, and Yutian County People’s Hospital. By using the unique resident health file number as an identifier to match follow-up data with health examination data, we created a multidimensional integrated dataset that includes information on patients’ lifestyles, physiological indicators, and biochemical markers.

### Ethics statement

The study protocol was approved by the Medical Ethics Committee of Xinjiang Medical University (Approval No. XJYKDKR20220725002) on July 25, 2022. The data for this study was accessed from January 1, 2015, to December 31, 2023. After obtaining the data, the authors did not have access to personally identifiable information of individual participants, as all data were anonymized and de-identified to ensure compliance with ethical standards and privacy protection requirements. As this was a retrospective study utilizing anonymized data, the requirement for informed consent was waived by the ethics committee. All procedures were conducted in compliance with the ethical standards outlined in the Declaration of Helsinki and relevant national and institutional guidelines applicable at the time the cohort was established.

### Inclusion and exclusion criteria

Prior to data collection, all relevant personnel from the participating institutions underwent standardized training on data extraction forms and operational procedures to ensure consistency and accuracy in data collection. Medical records for all enrolled patients were reviewed by experienced researchers to verify the completeness and validity of key variables. To minimize bias due to differences in laboratory testing units, all test data were standardized before analysis. Obvious outliers (e.g., a height recorded as 1.45 cm) were flagged and rechecked, with final judgment made by the principal investigators or designated physicians. Unreasonable values were excluded after confirmation.

#### Inclusion criteria

(1) Age between 20 and 90 years; (2) Continuous and complete diabetes management records and follow-up data from 2015 to 2023; (3) Availability of concurrent health examination data, which could be accurately matched to follow-up data using the health file number; (4) Diagnosis of Type 2 Diabetes Mellitus (T2DM) based on the “Chinese Type 2 Diabetes Prevention and Treatment Guidelines”, with diagnostic criteria including: fasting plasma glucose (FPG) ≥7.0 mmol/L, or a 2-hour post-oral glucose tolerance test (OGTT) glucose level ≥11.1 mmol/L, or current use of antidiabetic medication, or a documented history of diabetes.

#### Exclusion criteria

(1) Non-T2DM patients, such as those with Type 1 Diabetes, gestational diabetes, or other special types; (2) Incomplete follow-up data, making it impossible to assess the study outcomes; (3) Insufficient clinical information in medical records, making accurate risk modeling impossible; (4) Use of specific medications that may directly impact the risk of macrovascular disease; (5) Presence of macrovascular complications (e.g., coronary artery disease, stroke, or peripheral artery disease) at baseline; (6) Diagnosis of malignant tumors, chronic infectious diseases, autoimmune diseases, psychiatric or neurological disorders, severe heart disease, or severe kidney disease.

### Sample size calculation

This study used power analysis to calculate the sample size. Based on the design objectives of this study, we selected commonly used sample size calculation formulas, estimating the sample size based on effect size, significance level, and statistical power. For predicting whether diabetic patients will develop macrovascular complications, the basic formula for sample size calculation is as shown in Equation 1.

(1) $$n=\frac{{\left(Z_{a/2}+Z_{\beta }\right)}^{2}\cdot P(1-P)}{d^{2}},$$

Where:

*n*: Required sample size;

$Z_{a/2}$: Critical value of the standard normal distribution corresponding to the significance level;

$Z_{\beta }$: Critical value of the standard normal distribution corresponding to statistical power;

*d*: Acceptable margin of error (i.e., effect size), with 0.05 typically chosen as the tolerable error;

*P*:Expected classification probability (e.g., the proportion of healthy vs. unhealthy). Assuming equal probabilities for health and illness, *P* = 0.5.

Based on the sample size calculation formula, the required sample size to ensure 80% statistical power and a 5% significance level is approximately 784. In the actual data, we used 4,186 valid records.

### Definition of disease diagnosis criteria

The diagnostic criteria for Type 2 Diabetes Mellitus (T2DM) are based on the “Chinese Type 2 Diabetes Prevention and Treatment Guidelines (2023 Edition)” (36). T2DM can be diagnosed if the first criterion is met, along with one of the following conditions: (1) typical diabetes symptoms (polydipsia, polyuria, polyphagia, unexplained weight loss); (2) random venous plasma glucose ≥11.1 mmol/L; (3) fasting venous plasma glucose ≥7.0 mmol/L; or (4) plasma glucose ≥11.1 mmol/L 2 hours after an oral glucose tolerance test (OGTT).

Fasting state refers to at least 8 hours without caloric intake, and random blood glucose refers to any measurement of blood glucose at any time of the day without considering the time of the last meal. Random blood glucose cannot be used to diagnose abnormal fasting glucose or impaired glucose tolerance. If typical symptoms are absent, follow-up testing is required to confirm the diagnosis with fasting venous plasma glucose or 2-hour post-load plasma glucose levels.

### Diagnostic criteria for the diagnosis of macrovascular complications in T2DM

The diagnostic criteria for macrovascular complications of T2DM are based on the “Chinese Type 2 Diabetes Prevention and Treatment Guidelines (2023 Edition)” and the “Expert Consensus on the Diagnosis and Treatment of Cardiovascular Disease in Diabetic Patients” (37). T2DM complications are classified into acute and chronic complications. Acute complications include diabetic ketoacidosis, hyperosmolar non-ketotic diabetic coma, and lactic acidosis or hypoglycemic coma occurring during diabetes treatment. Chronic complications include macrovascular complications (cerebrovascular disease, cardiovascular disease, large artery atherosclerosis), microvascular complications (diabetic nephropathy, diabetic retinopathy, diabetic neuropathy), and diabetic foot. In this study, T2DM+C refers to diabetes with macrovascular complications, including cerebrovascular disease, cardiovascular disease, and large artery atherosclerosis.

### Statistical methods

The initial dataset included 310 variables. After removing irrelevant or incomplete data, 52 variables were retained, covering demographic, clinical, physiological, and lifestyle characteristics.

Patient data were categorized into continuous and categorical variables. Continuous variables with normal distribution were expressed as mean ± standard deviation (± SD), while non-normally distributed variables were presented as median (interquartile range, M[IQR]). Categorical variables were summarized as frequency (proportion, n[%]). For group comparisons, independent sample t-tests were applied to normally distributed continuous variables, Mann-Whitney *U* tests to non-normally distributed continuous variables, and *χ*² or Fisher’s exact tests to categorical variables. A significance level of *P* < 0.05 was considered. Statistical analyses were performed using SPSS software version 25.0.

The study compared multiple machine learning models, including AdaBoost, Random Forest, Extremely Randomized Trees, GBDT, Logistic Regression, MLP, SVM, and XGBoost, each with unique advantages for different datasets and tasks (38). All models were implemented in Python, developed, debugged, and optimized in PyCharm 2024.1.7. Data splitting was performed using stratified k-fold cross-validation from scikit-learn. To address class imbalance, the training set was balanced with the SMOTEENN method, combining over-sampling and under-sampling techniques to enhance model performance. The model hyperparameters were optimized via a 10-fold cross-validated grid search using the GridSearchCV function from the Python scikit-learn library, with the AUC-ROC as the metric for selecting the optimal parameter set.

Performance evaluation metrics included Accuracy, Sensitivity, Specificity, Precision, F1 Score, G-mean, Matthews Correlation Coefficient (MCC), and Area Under the Curve (AUC). To prevent overfitting, ten-fold cross-validation (10-fold Cross-Validation) was used. The importance of clinical features was assessed through Shapley Additive Explanations (SHAP) scores. The diagnostic performance of the models was evaluated using Area Under the Receiver Operating Characteristic Curve (AUROC), sensitivity, specificity, Positive Predictive Value (PPV), and Negative Predictive Value (NPV), and further validated for clinical applicability through Decision Curve Analysis (DCA).They form a comprehensive evaluation framework that spans overall model performance, class balance robustness, statistical reliability, and clinical utility.

## Results

### Inclusion process and overview of the study population

**Figure 1:**
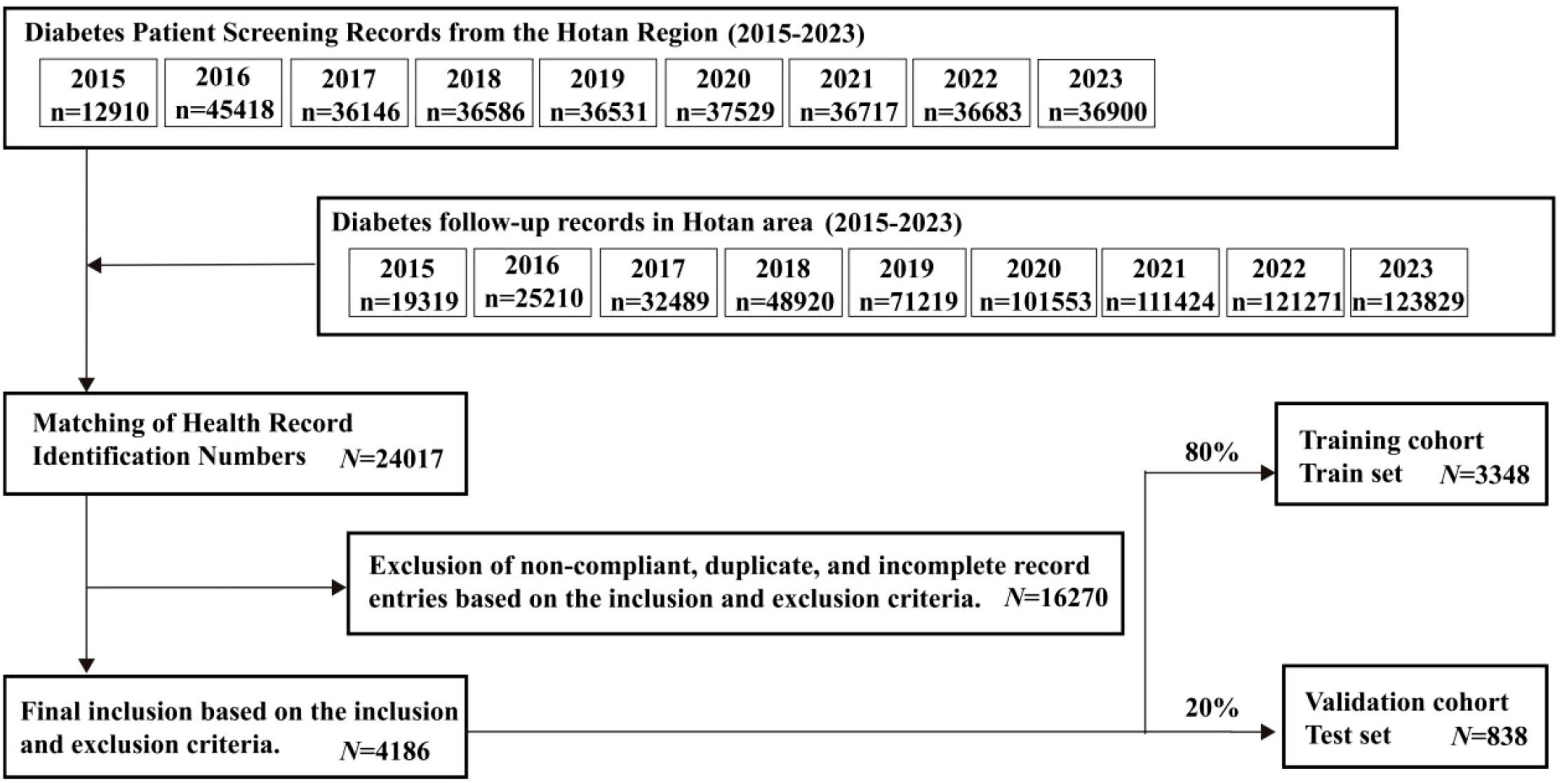
Inclusion process and overview of the study population.

This study used diabetes check-up and follow-up records from the Hotan region (2015-2023), as shown in **Figure 1**. The initial dataset included 454,418 check-up and 589,759 follow-up records, which were matched to yield 24,017 health records. After excluding 16,270 incomplete or invalid records, 4,186 eligible records remained. These were randomly divided into a training set (3,348 records) and a validation set (838 records) for developing and validating a prediction model for diabetic patients’ health status and follow-up conditions. The data processing involved matching check-up and follow-up records by health file number and retaining the earliest entry for consistency. Finally, 80% of the valid records were used for training, and 20% for validation.

### General information about the research subject

A total of 4,186 participants were included in this study, comprising 2,285 males (54.6%) and 1,901 females (45.4%). The mean age of the participants was 60.49 ± 8.38 years. There was no statistically significant difference in age between males and females (*P* = 0.22), nor in the distribution across different age groups (*P* = 0.87). The majority of participants were registered local residents (98.6%), with no gender difference observed in regional distribution (*P* = 0.72). A significant gender difference was found in marital status (*P* < 0.001), with the proportion of unmarried/divorced males (16.0%) being significantly higher than that of females (4.3%). The overall cumulative prevalence rate was 11.5%, with male and female prevalence rates being 11.5% and 11.3%, respectively; this difference was not statistically significant (*P* = 0.84), as shown in **Table 1**.

**Table 1:** The comparison of gender differences in demographic characteristics and prevalence of the study subjects.

Factor	Total cohort (*N* = 4186)	Male (n = 2285)	Female (n = 1901)	$\chi^{2}/t$ /F/U	*P-value*
Age( $\bar{X}$ ± *S*)	60.49 ± 8.38	60.63 ± 8.08	60.32 ± 8.73	1.23	0.22
Age Group, *n*(%)				1.23	0.87
<=50	356(8.5%)	169(7.3961%)	187(9.8369%)		
<=60	1899(45.4%)	1052(46.039%)	847(44.555%)		
<=70	1356(32.4%)	768(33.611%)	588(30.931%)		
>70	575(13.7%)	296(12.954%)	279(14.676%)		
Ethnicity(categorical)				0.65	0.72
Uighur nationality	58(1.3%)	27(1.1%)	31(1.6%)		
Other	4128(98.6%)	2258(98.8%)	1870(98.3%)		
Marital, *n*(%)				257.54	<0.001
Married	3737(89.2%)	1919(83.9%)	1818(95.6%)		
Single/divorced	449(10.7%)	366(16.0%)	83(4.3%)		
Number of cases(%)	481(11.5%)	265(11.5%)	216(11.3%)	0.04	0.84

### The comparison of demographic characteristics between the T2DM and T2DM+C groups

The results showed a clear trend toward younger patients in the T2DM+C cohort, with 22.9% of patients aged ≤50 years, significantly higher than the 6.6% in the pure T2DM cohort (*χ*²=217.61, *P* < 0.001). In contrast, variables such as gender, regional distribution, and marital status did not show statistically significant differences between the two groups (*P*>0.05), as shown in **Table 2**.

**Table 2:** Comparison of the demographic characteristic distribution between pure T2DM and T2DM+C.

Factor	Total cohort (*N* = 4186)	T2DM cohort (n = 3705)	T2DM+C cohort (n = 481)	$\chi^{2}/t$ /F/U	*P-value*
Age( $\bar{X}$ ± *S*)	60.49 ± 8.38	60.87 ± 8.07	57.54 ± 10.02	0.94	0.3475
Age Group, *n*(%)				217.61	<0.001
<=50	356(8.5%)	246(6.6%)	110(22.9%)		
<=60	1899(45.4%)	1704(46.0%)	185(40.5%)		
<=70	1356(32.4%)	1087(33.2%)	125(26.0%)		
>70	575(13.7%)	475(14.1%)	51(10.6%)		
Sex, *n*(%)				0.282	0.595
Male	2285(54.5%)	2020(54.5%)	265(55.0%)		
Female	1901(45.4%)	1685(45.4%)	216(49.9%)		
Ethnicity(categorical)				0.0565	0.811
Uighur nationality	58(1.3%)	52(1.4%)	6(1.2%)		
Other	4128(98.6%)	3653(98.5%)	475(98.7%)		
Marital, *n*(%)				0.3978	0.528
Married	3737(89.2%)	3313(89.4%)	424(88.1%)		
Single/divorced	449(10.7%)	392(10.5%)	57(11.8%)		

Gender: 0 for male, 1 for female; Age: 1 for ≤50 years, 2 for ≤60 years, 3 for ≤70 years, 4 for >70 years; Region: 0 for outsider, 1 for local; Marital status: 0 for married, 1 for single/divorced.

### Comparison of physical examination indicators between T2DM and T2DM+C

It was found that the waist circumference of this cohort was significantly higher than that of the pure T2DM group (median 97 [89–106] cm vs. 93 [85–100] cm, *P* < 0.001). Additionally, the incidence of oropharyngeal abnormalities was higher (1.0% vs. 0.2%, *P* = 0.012), and the proportion of patients with absent lung crackles was also higher (100% vs. 99.2%, *P* < 0.001). However, no statistically significant differences were found between the two groups in terms of hypoglycemic response, heart rate, liver and spleen enlargement, or other physiological indicators (*P*>0.05), as shown in **Table 3**.

**Table 3:** Comparison of physiological indicators between the pure T2DM cohort and the T2DM+C cohort.

Factor	Total cohort (*N* = 4186)	T2DM cohort (n = 3705)	T2DM+C cohort (n = 481)	$\chi^{2}/t$ /F/U	*P-value*
Lower Extremity Edema, *n*(%)				159.21	<0.001
No	4160(99.4%)	3682(99.3%)	478(99.3%)		
Yes	26(0.6%)	70(0.6%)	3(0.6%)		
Hypoglycemic Reaction, *n*(%)				0.5155	0.473
No	4103(98.1%)	3635(98.1%)	468(97.2%)		
Yes	83(1.8%)	83(1.8%)	13(2.7%)		
Hearing Ability, *n*(%)				0.548	0.4597
Normal	4123(98.4%)	3647(98.4%)	476(98.9%)		
Impaired	63(1.5%)	58(1.5%)	5(1.0%)		
RR(/min)( $\bar{X}$*± S*)	20.04 ± 3.17	20.05 ± 3.15	19.93 ± 3.36	0.5979	0.43943
Pharyngeal Throat, *n*(%)				13.76	0.0115
Normal	4173(99.6%)	3697(99.7%)	476(98.9%)		
Impaired	13(0.3%)	8(0.2%)	5(1.0%)		
Scleral Check, *n*(%)				1.334	0.4639
Normal	4173(99.5%)	3689(99.5%)	480(99.7%)		
Impaired	17(0.4%)	16(0.4%)	1(0.2%)		
HR(/min)( $\bar{X}$ ± *S*)	78(71, 85)	78(71, 85)	79(72, 86)	2.1107	0.1464
Hepatomegaly, *n*(%)				4.195	0.6084
No	4178(99.7%)	3697(99.2%)	481(100%)		
Yes	8(0.2%)	8(0.7%)	0(0%)		
Lung Breath Sounds, *n*(%)				0.482	0.707
Normal	4156(99.2%)	3678(99.2%)	478(99.3%)		
Impaired	30(0.7%)	27(0.7%)	3(0.6%)		
Rales, *n*(%)				21.59	<0.001
No	4156(99.3%)	3679(99.2%)	481(100%)		
Yes	26(0.6%)	26(0.7%)	0(0%)		
PR(/min)	80 (73, 86)	80 (73, 86)	80 (74, 88)	1.5172	0.2181
Splenomegaly, *n*(%)				6.287	0.204
No	4179(99.7%)	3698(99.8%)	481(100%)		
Yes	7(0.2%)	7(0.1%)	0(0%)		
WC(cm)( $\bar{X}$ ± *S*)	94(85, 100)	93(85, 100)	97(89, 106)	45.8134	<0.001
Abdominal Mass, *n*(%)				0.442	0.4334
No	4175(99.6%)	3694(99.7%)	481(100%)		
Yes	11(0.3%)	11(0.2%)	0(0%)		
Abdominal Tenderness, *n*(%)				1.757	0.3037
No	4155(99.2%)	3675(99.1%)	480(99.7%)		
Yes	31(0.7%)	30(0.8%)	1(0.2%)		
Barrel Chest, *n*(%)				0.312	0.546
No	4172(99.6%)	3691(99.6%)	481(100%)		
Yes	14(0.3%)	14(0.3%)	0(0%)		

Numerical values are represented as median (lower quartile - upper quartile). In the table, categorical variables are assigned default values with the first group as 0 and the second group as 1, such as Hearing Ability (0: Normal, 1: Impaired); Normal; Impaired. RR, Respiratory Rate; HR, Heart Rate; PR, Pulse Rate; WC, Waist Circumference.

### Comparison of biochemical indicators between T2DM and T2DM+C

The results revealed significant differences between this cohort and the pure T2DM group in multiple physiological indicators. Specifically, the BMI of the T2DM+C group was significantly higher (*Z* = 3.25, *P* < 0.001). Liver function markers, such as ALT, also showed an upward trend (*Z* = 2.30, *P* = 0.02). However, fasting blood glucose levels paradoxically decreased (*Z* = 4.21, *P* < 0.001). In addition, the T2DM+C group exhibited borderline abnormalities in blood urea (*Z* = 2.15, *P* = 0.03) and AST (*Z* = 1.97, *P* = 0.05), indicating a potential risk of multi-organ interactive damage. However, traditional cardiovascular risk factors such as blood lipids and blood pressure did not show significant differences between the two groups (all *P*>0.05), as shown in **Table 4**.

**Table 4:** Comparison of biochemical indicators between the T2DM-only cohort and the T2DM+C (with complications) cohort.

Factor	Total cohort (N = 4186)	T2DM cohort (n = 3705)	T2DM+C cohort (n = 481)	*Z*	*P-value*
BT(°C)	36.3(36.2, 36.5)	36.3(36.2, 36.5)	36.4(36.2, 36.5)	1.34	< 0.001
BMI(kg/m²)	26.60(24.40,38.6)	26.48(24.24,28.3)	28.48(25.70, 31.24)	3.25	< 0.001
DBP (mmHg)	75.0(67.5, 85.0)	75.0(67.5, 85.0)	75.0(66.38, 85.00)	0.89	0.37
SBP(mmHg)	130(120, 136)	130(120, 135)	130(120, 140)	1.12	0.26
TC(mmol/L)	4.56(3.81, 5.41)	4.60(3.85, 5.44)	4.34(3.60, 5.16)	0.72	0.47
TBIL(µmol/L)	11.93(8.70, 15.60)	11.90(8.64, 15.60)	12.30(9.25, 15.32)	0.60	0.55
TG(mmol/L)	1.59(1.12, 2.38)	1.58(1.12, 2.38)	1.63(1.11, 2.40)	0.99	0.32
WBC(10^9^/L)	6.88(5.80, 8.10)	6.89(5.78, 8.10)	6.83(5.93, 8.14)	1.01	0.31
ALB(g/L)	44.0(41.3, 46.7)	44.10(41.50,46.7)	42.90(40.25, 46.42)	1.20	0.23
FBG(mmol/L)	7.62(5.48, 11.73)	7.69(5.48, 11.80)	7.12(5.45, 11.09)	4.21	< 0.001
DBIL(µmol/L)	3.60(2.50, 5.30)	3.60(2.50, 5.24)	3.62(2.60, 5.50)	1.56	0.12
PLT(10^9^/L)	246(207, 290)	246(207, 289)	247(205.75, 293)	0.87	0.39
BU(mmol/L)	4.93(3.96 6.03)	4.94(3.98 6.06)	4.78(3.84 5.80)	2.15	0.03
LDL-C(mmol/L)	2.60(2.02, 3.30)	2.63(2.04, 3.34)	2.63(1.97, 3.03)	1.30	0.19
SCr(µmol/L)	58.9(46.0, 73.2)	58.80(46.00,73.2)	59.00(45.22, 73.93)	0.78	0.44
ALT(U/L)	22.1(15.9, 31.5)	22.00(15.70,31.1)	24.00(17.00,33.11)	2.30	0.02
AST(U/L)	20.3(16.0, 26.5)	20.04(16.0, 26.20)	22.00(16.78,28.25)	1.97	0.05
HGB(g/L)	146(135, 158)	146(135, 158)	147(136, 158)	0.56	0.57
HT(cm)	160(155, 166)	160(155, 166)	160(154.00,166.25)	0.12	0.91

Biochemical indicators are presented as median (lower quartile - upper quartile). BT, Body Temperature; BMI, Body Mass Index; DBP, Diastolic Blood Pressure; SBP, Systolic Blood Pressure; RR, Respiratory Rate; HR, Heart Rate; TC, Total Cholesterol; TBIL, Total Bilirubin; TG, Triglycerides; WBC, White Blood Cells; ALB, Albumin; FBG, Fasting Blood Glucose; DBIL, Direct Bilirubin; PR, Pulse Rate; WC, Waist Circumference; PLT, Platelets; BU, Blood Urea; LDL-C, Low-Density Lipoprotein Cholesterol; SCr, Serum Creatinine; ALT, Alanine Aminotransferase; AST, Aspartate Aminotransferase; HGB, Hemoglobin; HT, Height.

### Comparison of lifestyle follow-up indicators between T2DM and T2DM+C

Daily smoking amount (*P* < 0.05) and alcohol consumption (*P* < 0.05) were significantly higher than the control group, while weekly exercise frequency was significantly lower (*P* < 0.05). Notably, despite these objective behavioral risk differences, the proportion of the T2DM+C group self-reporting their physical health status as “poor” showed a reverse trend (*P* < 0.05). There were no statistically significant differences between the two groups in psychological adjustment levels, exercise duration per session, and other indicators (all *P*>0.05). A potential disconnect between health behavior risks and subjective health perceptions in this population (**Table 5**).

**Table 5:** Comparison of lifestyle characteristics between the T2DM and T2DM+C cohorts.

Factor	Total cohort (*N* = 4186)	T2DM cohort (n = 3705)	T2DM+C cohort (n = 481)	$\chi^{2}/t$ /F/U	*P-value*
Years of Exercise	0.17 ± 1.37	0.18 ± 1.36	0.12 ± 1.41	0.89	0.15
Smoking Status, *n*(%)				1.22	0.45
Non-smokers	3937 (94.3%)	3490(94.1%)	447(92.9%)		
Smokers	249(5.9%)	215(5.8%)	34(7.0%)		
Daily Smoking Consumption	0.74 ± 3.83	0.68 ± 3.52	1.16 ± 5.66	2.32	0.01
Drinking Frequency, *n*(%)				0.53	0.65
None	4074 (97.3%)	3603(97.3%)	466(96.8%)		
Yes	112(2.7%)	97(2.6%)	15(3.1%)		
Daily Drinking Consumption	1.41 ± 52.72	0.56 ± 12.01	7.99 ± 151.90	2.11	0.02
Per Exercise Time(/min)	1.31 ± 7.29	1.37 ± 7.41	0.85 ± 6.25	0.07	0.07
Exercise Duration	11.92 ± 15.51	12.36 ± 15.62	8.56 ± 14.20	1.43	0.12
(/min)					
Exercise Frequency (week/times)	2.03 ± 2.54	2.13 ± 2.58	1.23 ± 2.03	3.21	0.02
Exercise Frequency (times/week)	1.13 ± 0.57	1.12 ± 0.55	1.19 ± 0.70	0.8	0.8
Psychological Adjustment, *n*(%)				2.93	0.12
good	4152(99.2%)	3679(99.2%)	473(98.3%)		
Poor	34(0.8%)	26(0.7%)	8(1.6%)		
Physical Functional Status, *n*(%)				4.65	0.03
Good	4151(99.2%)	3673(99.1%)	478(99.3%)		
Poor	35(0.8%)	32(0.8%)	3(0.6%)		

The data in the table are described by ( $\bar{X}$*± S*) mean ± standard deviation. The value of categorical variables is assigned to 0 in the first row and 1 in the second row by default. Daily Smoking Consumption (cigarettes/day), Daily Drinking Consumption (units/day), and Exercise Duration (minutes).

### Dataset partitioning and baseline characteristics

The total cohort included 4186 participants (54.5% male), with 63.2% aged ≤60 years. To ensure the reliability of predictive modeling, the population was randomly divided into a training set (n = 3348) and a validation set (n = 838). Chi-square tests confirmed no significant differences in key demographic and clinical indicators, including sex (*P* = 0.865), residency (*P* = 0.627), and macrovascular complication prevalence (P = 0.920), indicating appropriate covariate balance (**Supplementary Table S1**).

Clinical and biochemical variables were also well balanced across sets. BMI averaged 26.53 kg/m², slightly higher in the validation group. Mean DBP and SBP were 76.6 mmHg and 129.3 mmHg, respectively. Levels of TC, TBIL, and TG remained consistent between groups, with minimal deviation. These results further support the validity of the dataset division for model development (**Supplementary Table S2**).

Lifestyle characteristics, including smoking (5.9%–6.2%), weekly exercise frequency (~2.0 times), and family history (4.2%–4.9%), demonstrated strong consistency. While alcohol intake was modestly elevated in the validation group, other indicators such as exercise duration and psychological adjustment remained stable. Overall, the partitioned datasets exhibited comparable lifestyle profiles (**Supplementary Table S3**).

### Shapley additive explanations of key predictive factors for 8 machine learning methods

**Figure 2:**
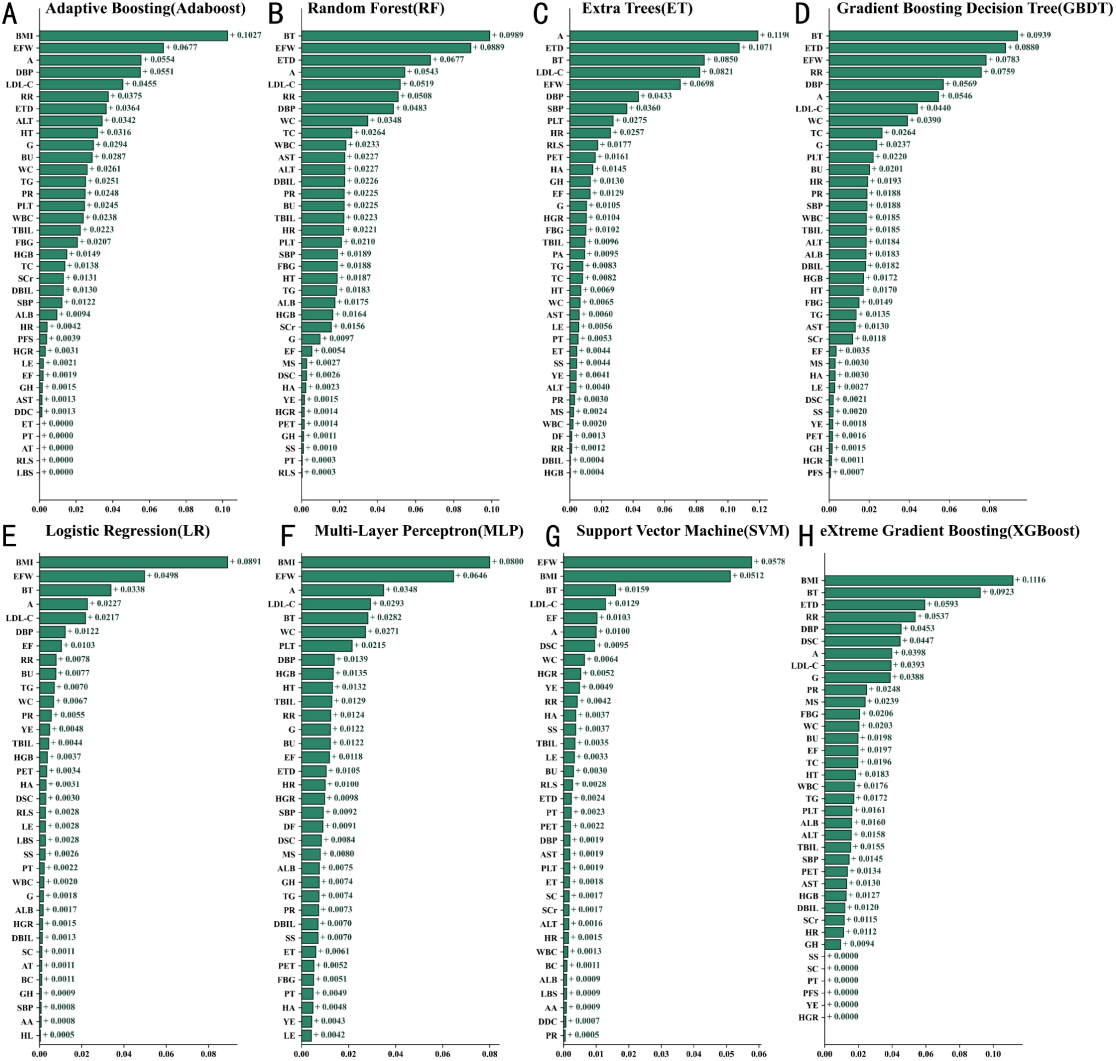
Comparison of feature contributions in machine learning models based on SHAP **(A)** XGBoost **(B)** Random Forest **(C)** AdaBoost **(D)** Gradient Boosting Decision Trees **(E)** Logistic Regression **(F)** Multi-Layer Perceptron **(G)** Support Vector Machine **(H)** Extreme Gradient Boosting. Biochemical and physiological indicators are presented as median (lower quartile – upper quartile). A, Age; AA, Abdominal Mass; ALB, Albumin; ALT, Alanine Aminotransferase; AST, Aspartate Aminotransferase; AT, Abdominal Tenderness; BC, Barrel Chest; BMI, Body Mass Index; BT, Body Temperature; BU, Blood Urea; DBIL, Direct Bilirubin; DBP, Diastolic Blood Pressure; DDC, Daily Drinking Consumption; DF, Drinking Frequency; DSC, Daily Smoking Consumption; EF, Exercise Frequency; EFW, Exercise Frequency per Week; ET, Encompassing Territory; ETD, Exercise Duration; FBG, Fasting Blood Glucose; G, Gender; GH, Genetic History; HA, Hearing Ability; HGB, Hemoglobin; HL, Hepatomegaly; HR, Heart Rate; HT, Height; HGR, Hypoglycemic Reaction; LBS, Lung Breath Sounds; LDL-C, Low-Density Lipoprotein Cholesterol; LE, Lower Extremity Edema; LNC, Lymph Node Check; MS, Marital Status; PA, Psychological Adjustment; PFS, Physical Functional Status; PET, Per Exercise Time; PLT, Platelets; PR, Pulse Rate; PT, Pharyngeal Throat; RLS, Rales; RR, Respiratory Rate; SBP, Systolic Blood Pressure; SC, Scleral Check; SCr, Serum Creatinine; SL, Splenomegaly; SS, Smoking Status; TBIL, Total Bilirubin; TC, Total Cholesterol; TG, Triglycerides; VDEO, Vascular Disease Endpoint Outcome; WBC, White Blood Cells; WC, Waist Circumference; YE, Years of Exercise.

A total of 52 variables were integrated from the training set into the ML algorithms to construct an initial model. SHapley Additive Explanations (SHAP) analysis was then used to assess variable importance, ranking the feature contributions in predicting macrovascular complications of T2DM across various machine learning models, as shown in **Figure 2**. The XGBoost model was ultimately selected as the prediction tool based on its superior performance (**Figure 2H**). This model incorporated 32 key predictors, including clinical indicators such as body mass index (BMI), body temperature (BT), respiratory rate (RR), diastolic blood pressure (DBP), low-density lipoprotein cholesterol (LDL-C), and fasting blood glucose (FBG). Feature importance analysis revealed BMI and BT consistently ranked as the top two factors, with age (A), exercise frequency (EF), and systolic blood pressure (SBP) also showing strong predictive value. SHAP analysis further confirmed BMI (SHAP value +0.1116), BT (SHAP value +0.0923), and LDL-C (SHAP value +0.0821) as the most influential predictors.

### SHAP feature contribution analysis of the XGBoost model

**Figure 3:**
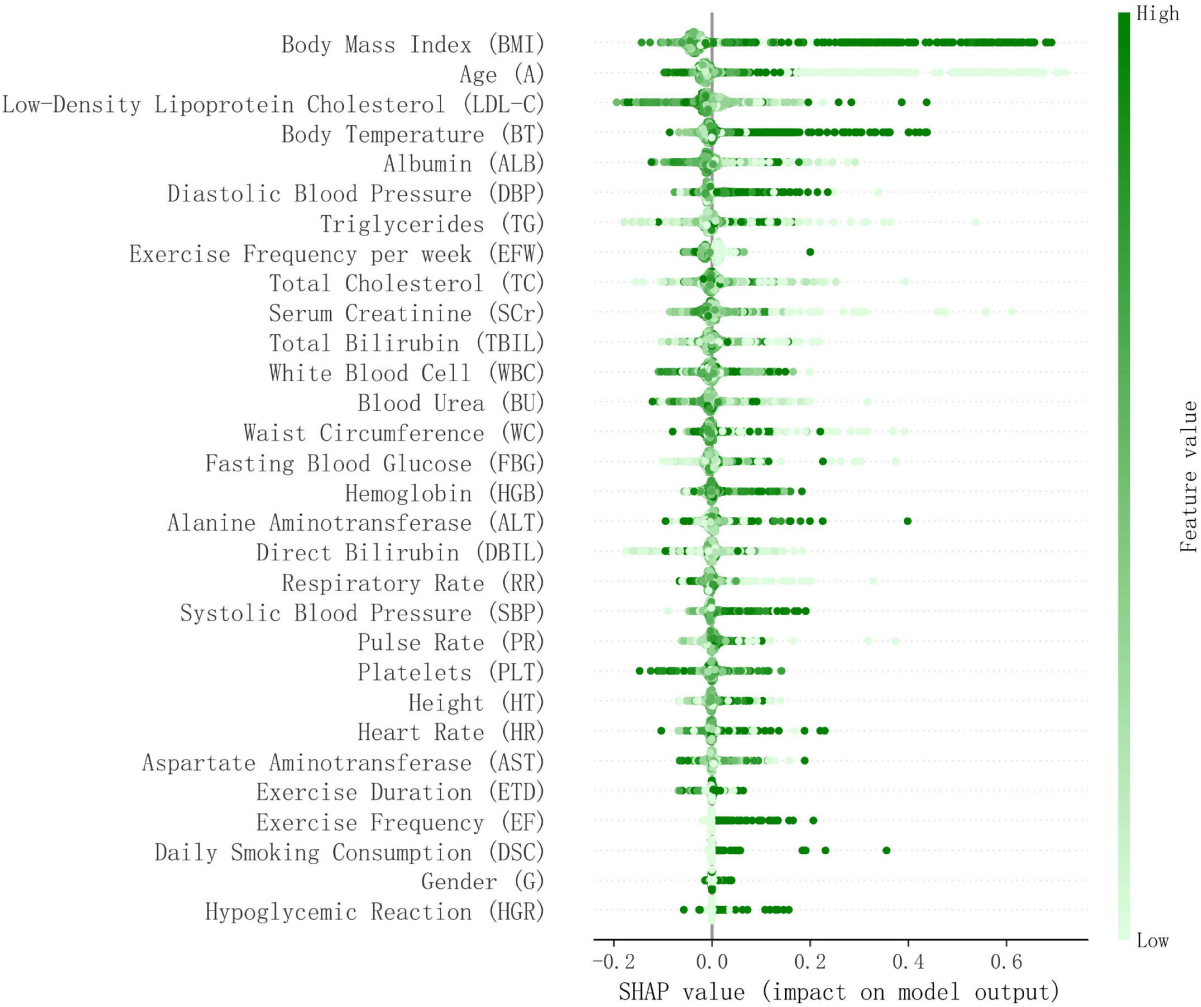
SHAP-based feature contribution analysis of the XGBoost model (bee swarm plot).

**Figure 3**. SHAP Values Quantify the Impact and Direction of Different Features on Model Predictions. The analysis revealed that body mass index (BMI), age, and low-density lipoprotein cholesterol (LDL-C) had significant predictive power for disease risk. Specifically, higher BMI was strongly positively correlated with the occurrence of macrovascular diseases, while increased age and elevated LDL-C levels also significantly heightened the risk of disease. Elevated body temperature was found to be closely associated with an increased risk of complications, likely due to alterations in metabolic status. Decreased albumin levels and low diastolic blood pressure were also identified as risk-enhancing factors, highlighting the importance of nutritional status and circulatory system function. Notably, although factors such as gender differences, hypoglycemic reactions, and daily smoking amount contributed less to the model’s predictions, their inclusion enhanced the model’s ability to recognize complex patterns in clinical data.

### Discriminatory performance evaluation of machine learning models

**Figure 4:**
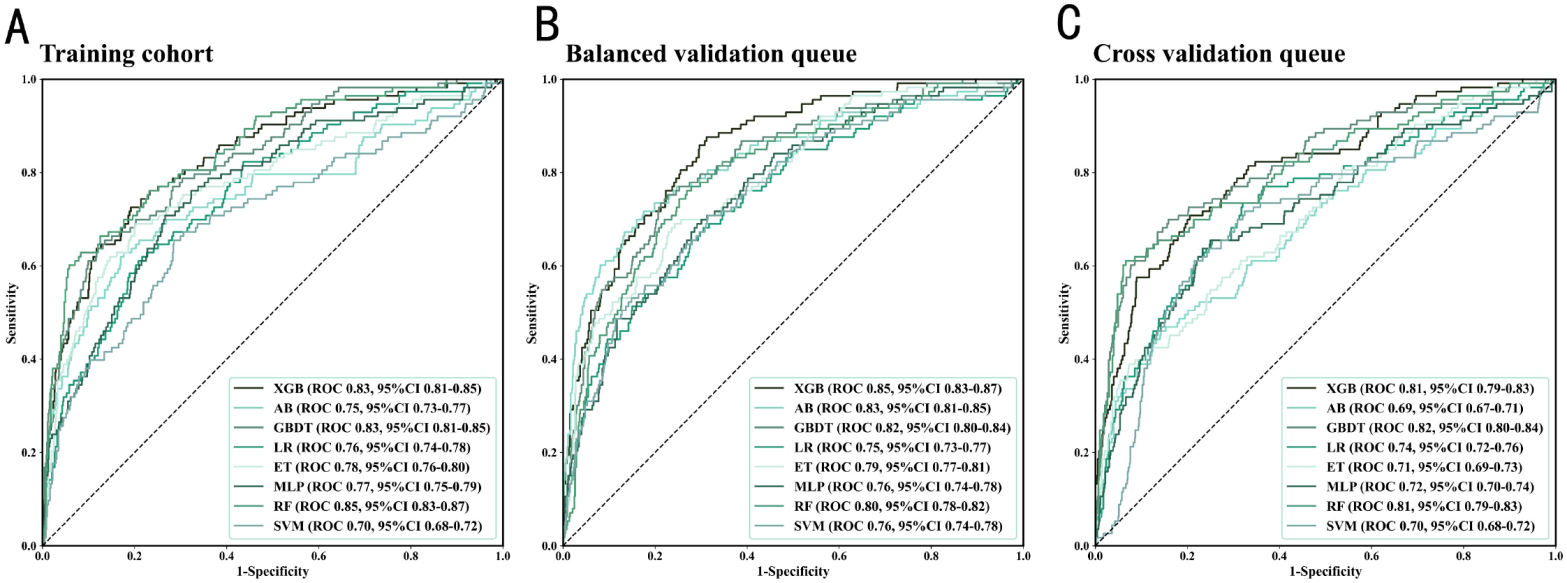
Performance comparison of T2DM macrovascular complication diagnosis models across training, balanced test, and cross-validation datasets.

This study evaluated the performance of eight machine learning models in predicting macrovascular complications of T2DM. All models achieved AUC values between 0.68 and 0.85 on the test set (**Figure 4A**), with the XGBoost model performing the best. In the balanced and validation cohorts, the AUROC ranges were 0.74–0.87 (**Figure 4B**) and 0.68–0.83 (**Figure 4C**), respectively, confirming the models’ good generalizability. To ensure the reliability of the evaluation results, a 10-fold nested cross-validation approach was employed, with validation conducted through 100 iterations and averaged values. As shown in **Table 6**, in the training cohort, the XGBoost model achieved an accuracy of 0.88578, slightly lower than random forest (0.88730), but significantly outperforming the other models. This model exhibited high specificity (0.97318), indicating its ability to effectively identify non-complicated cases, while maintaining a moderate sensitivity (0.32354). In the balanced validation cohort, the XGBoost model’s accuracy was 0.84121, with sensitivity increasing to 0.58884, demonstrating good adaptability. Cross-validation results further confirmed the model’s stability, maintaining accuracy and specificity at 0.88503 and 0.97253, respectively. Apart from XGBoost, the GBDT model excelled in sensitivity (0.33549), while the AdaBoost model also showed good predictive performance.

**Table 6:** Diagnostic performance of ML models for T2DM macrovascular complications.

Model cohort	Accuracy	Sensitivity	Specificity	Precision	F1 score
Training cohort					
Adaboost	0.88 (0.88-0.88)	0.29 (0.28-0.30)	0.97 (0.97-0.97)	0.62 (0.61-0.63)	0.40 (0.39-0.41)
RF	0.89 (0.89-0.89)	0.23 (0.22-0.24)	0.99 (0.99-0.99)	0.76 (0.74-0.77)	0.35 (0.34-0.36)
ET	0.25 (0.24-0.25)	0.98 (0.98-0.98)	0.13 (0.13-0.13)	0.15 (0.15-0.15)	0.26 (0.26-0.27)
GBDT	0.89 (0.89-0.89)	0.34 (0.33-0.34)	0.98 (0.98-0.98)	0.71 (0.70-0.72)	0.45 (0.44-0.46)
LR	0.66 (0.66-0.66)	0.72 (0.72-0.73)	0.65 (0.65-0.65)	0.25 (0.24-0.25)	0.37 (0.36-0.37)
MLP	0.87 (0.87-0.87)	0.11 (0.10-0.11)	0.99 (0.99-0.99)	0.71 (0.69-0.73)	0.18 (0.18-0.19)
SVM	0.53 (0.53-0.53)	0.87 (0.86-0.87)	0.48 (0.48-0.48)	0.21 (0.20-0.21)	0.33 (0.33-0.34)
XGBoost	0.89 (0.88-0.89)	0.32 (0.31-0.33)	0.97 (0.97-0.97)	0.65 (0.64-0.66)	0.43 (0.42-0.44)
Balanced validation queue					
Adaboost	0.80 (0.80-0.80)	0.72 (0.71-0.73)	0.81 (0.81-0.81)	0.37 (0.36-0.38)	0.49 (0.48-0.49)
RF	0.73 (0.73-0.73)	0.75 (0.74-0.75)	0.73 (0.73-0.73)	0.30 (0.30-0.31)	0.43 (0.43-0.44)
ET	0.25 (0.24-0.25)	0.98 (0.98-0.98)	0.13 (0.13-0.13)	0.15 (0.15-0.15)	0.26 (0.26-0.27)
GBDT	0.78 (0.78-0.78)	0.68 (0.67-0.69)	0.80 (0.80-0.80)	0.35 (0.35-0.36)	0.46 (0.46-0.47)
LR	0.66 (0.66-0.66)	0.71 (0.71-0.72)	0.65 (0.65-0.65)	0.25 (0.24-0.25)	0.37 (0.36-0.37)
MLP	0.64 (0.63-0.64)	0.77 (0.76-0.78)	0.62 (0.61-0.62)	0.24 (0.23-0.24)	0.36 (0.36-0.37)
SVM	0.53 (0.53-0.53)	0.86 (0.86-0.87)	0.48 (0.48-0.48)	0.21 (0.20-0.21)	0.33 (0.33-0.34)
XGBoost	0.84 (0.84-0.84)	0.59 (0.58-0.60)	0.88 (0.88-0.88)	0.43 (0.43-0.44)	0.50 (0.49-0.50)
Cross validation queue					
Adaboost	0.88 (0.88-0.88)	0.29 (0.28-0.30)	0.97 (0.97-0.97)	0.62 (0.61-0.63)	0.40 (0.39-0.41)
RF	0.73 (0.73-0.73)	0.75 (0.75-0.76)	0.73 (0.73-0.73)	0.30 (0.30-0.31)	0.43 (0.43-0.44)
ET	0.25 (0.24-0.25)	0.98 (0.98-0.98)	0.13 (0.13-0.13)	0.15 (0.15-0.15)	0.26 (0.26-0.27)
GBDT	0.78 (0.78-0.78)	0.68 (0.67-0.69)	0.80 (0.80-0.80)	0.35 (0.34-0.36)	0.46 (0.46-0.46)
LR	0.66 (0.66-0.66)	0.71 (0.71-0.72)	0.65 (0.65-0.65)	0.24 (0.24-0.25)	0.36 (0.36-0.37)
MLP	0.63 (0.63-0.64)	0.76 (0.75-0.77)	0.61 (0.61-0.62)	0.23 (0.23-0.24)	0.36 (0.36-0.36)
SVM	0.53 (0.53-0.53)	0.87 (0.86-0.87)	0.48 (0.48-0.48)	0.21 (0.20-0.21)	0.33 (0.33-0.34)
XGBoost	0.89 (0.88-0.89)	0.32 (0.32-0.33)	0.97 (0.97-0.97)	0.65 (0.64-0.66)	0.43 (0.42-0.44)

### Decision curve analysis of machine learning models

**Figure 5:**
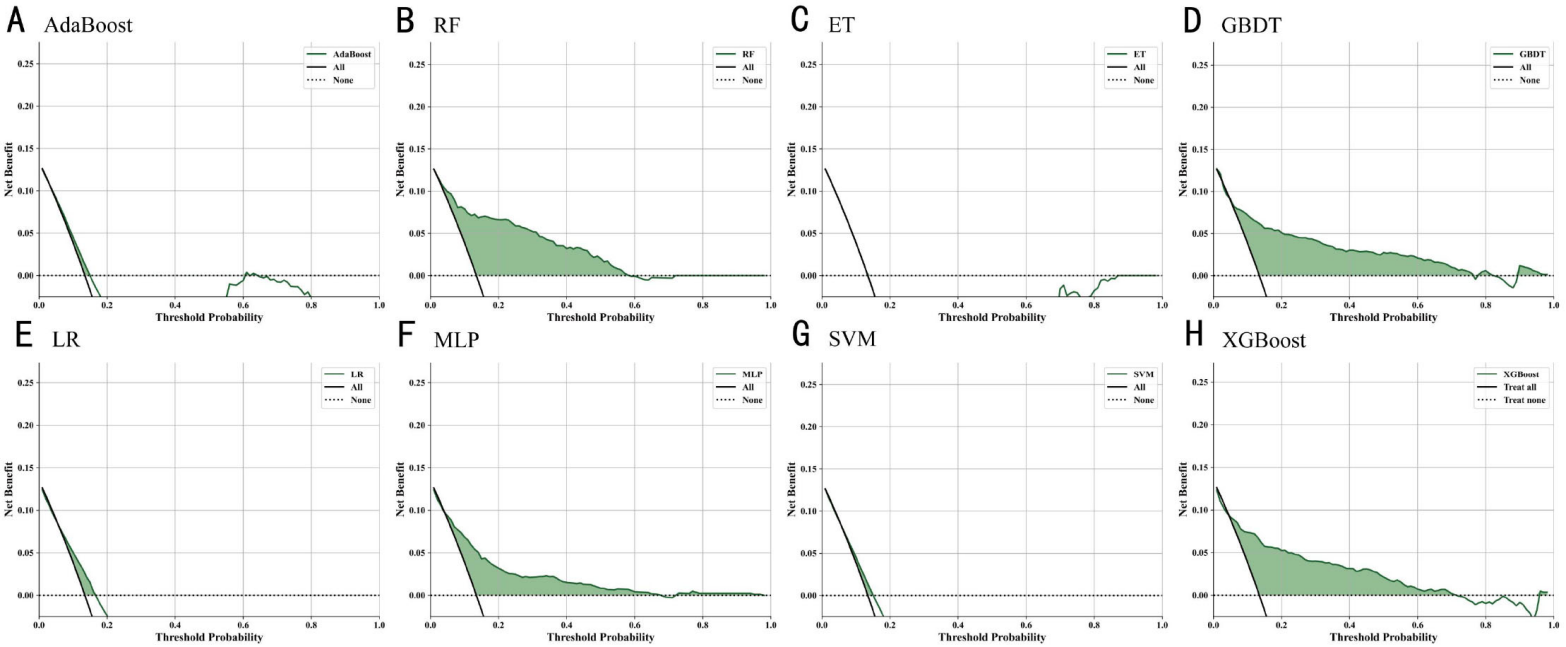
Decision curve analysis of ML models for diagnosing T2DM macrovascular complications: comparison of net benefit **(A)** XGBoost **(B)** Random Forest **(C)** AdaBoost **(D)** Gradient Boosting Decision Tree **(E)** Logistic Regression **(F)** Multi-Layer Perceptron **(G)** Support Vector Machine **(H)** Extreme Gradient Boosting.

As shown in **Figure 5**, the results demonstrated that Random Forest, XGBoost, MLP, and GBDT outperformed the “treat-all” and “treat-none” strategies across a wide range of threshold probabilities, indicating high clinical utility. The green shaded areas represent effective thresholds, highlighting these models’ advantages in identifying high-risk individuals. In contrast, AdaBoost, ET, LR, and SVM yielded lower net benefits, suggesting limited clinical applicability.

## Discussion

This study suggests that young T2DM patients (≤50 years) have a significantly higher risk of developing macrovascular complications, indicating that age may constitute an independent risk factor distinct from gender, region, and marital status.

T2DM patients with macrovascular disease generally exhibit more pronounced central obesity, suggesting that abnormal fat distribution may play a key role in the development of vascular complications. The release of inflammatory factors from adipose tissue is believed to trigger endothelial dysfunction and arteriosclerosis (39). Moreover, the phenotypic features of respiratory abnormalities, such as pharyngeal structural abnormalities and the disappearance of wheezing, may reflect disrupted respiratory compensation mechanisms associated with obesity, further exacerbating the cardiovascular burden (40).

In terms of metabolic parameters, T2DM+C patients exhibit characteristics of chronic inflammation, such as increased BMI and mild elevation in body temperature, which is consistent with previous research linking low-grade systemic inflammation to arteriosclerosis (41). Meanwhile, elevated liver enzymes suggest increased hepatic metabolic stress, while the abnormal decrease in fasting blood glucose may reflect late-stage pancreatic dysfunction or an enhanced treatment response. Additionally, small changes in renal function indicators, such as a decrease in blood urea nitrogen, may result from an early compensatory response in glomerular filtration, indicating an overall imbalance in the “metabolism-liver-kidney” axis. These changes collectively construct a model of complication progression driven by multi-organ interactions.

T2DM+C patients generally exhibit higher smoking, alcohol consumption, and physical inactivity rates, but their perception of their health status remains relatively low. This “high-risk behavior - low health risk perception” disconnect may stem from the latent early signs of macrovascular complications and the patients’ lack of understanding of the severity of chronic complications. Previous studies have pointed out that diabetic patients’ cognitive bias regarding their health significantly reduces their initiative and adherence to disease management (42). To address the disconnect between “high-risk behavior and low-risk perception”, science education is paramount. Additionally, establishing peer support groups where nursing teams facilitate experience sharing among patients who have successfully modified their behaviors leverages peer influence and social identity to boost confidence and motivation in new participants.

In-depth exploration using SHAP values identified several key factors influencing large vascular complications in T2DM patients. Body mass index (BMI) showed a significant positive impact, with higher values closely related to an increased risk. This finding is consistent with previous research linking obesity to large vascular diseases (43). Similarly, age emerged as a crucial risk factor, highlighting the role of aging in vascular degeneration (44). Elevated cholesterol levels were also strongly associated with large vascular diseases, further reinforcing the importance of cholesterol management in reducing cardiovascular risk (45, 46). Elevated cholesterol levels were also strongly associated with large vascular diseases, further reinforcing the importance of cholesterol management in reducing cardiovascular risk (31). Other findings include the role of triglycerides (TG), which were positively correlated with vascular diseases, consistent with previous studies emphasizing the cardiovascular risk associated with high triglyceride levels (47). In contrast, the frequency of weekly physical activity and the duration of each exercise session exhibited protective effects, confirming that regular physical activity can reduce the risk of large vascular complications (48). Total cholesterol and serum creatinine levels also showed positive correlations, emphasizing the contribution of dyslipidemia and kidney function impairment to vascular risk (49). Furthermore, higher fasting blood glucose levels were closely associated with increased risk, underscoring the critical role of blood sugar control in preventing complications (50, 51).

Compared to previous traditional models and machine learning models, the model proposed in this study demonstrates greater comprehensiveness and robustness by integrating 52 variable indicators that encompass lifestyle-related follow-up measures as well as clinical parameters of physiological and biochemical status. This approach extends the research scope, provides deeper predictive insights, and significantly enhances the reliability and applicability of the findings (52–54). Among them, the XGBoost model performed the best, achieving the highest accuracy, further confirming its advantage in type 2 diabetes-related research. Clinical utility evaluation revealed that models such as Random Forest, XGBoost, MLP, and GBDT provided higher net benefits across a broad range of threshold probabilities. Calibration analysis confirmed the reliability of these models, particularly XGBoost, whose predictions were highly consistent with actual observed results. These findings suggest that tree-based algorithms, especially XGBoost, offer robust and applicable tools for predicting large vascular complications and assisting clinical decision-making. This aligns with previous research, which demonstrated that Random Forest and XGBoost generally outperform other machine learning algorithms (55, 56).

BMI has consistently been one of the most important predictors of macrovascular complications in T2DM. Numerous studies have shown that a higher BMI is closely associated with an increased risk of cardiovascular diseases, including coronary artery disease, stroke, and other vascular events, with this relationship being particularly pronounced in the T2DM population. The mechanism can largely be attributed to insulin resistance caused by excessive fat accumulation, which is one of the main drivers of macrovascular disease development. Insulin resistance increases blood glucose levels and triglyceride levels, exacerbating atherosclerosis and endothelial dysfunction, thereby increasing the risk of cardiovascular diseases. Additionally, elevated BMI is not only associated with metabolic disorders but is also closely related to systemic inflammation and endothelial dysfunction, both of which are considered key mechanisms leading to vascular damage. Excessive fat accumulation, especially abdominal fat, promotes the secretion of various inflammatory factors from adipocytes, such as tumor necrosis factor-alpha (TNF-α) and interleukin-6 (IL-6), which can intensify the inflammatory response in the vascular wall and promote vascular wall thickening. Furthermore, an elevated BMI is closely linked to endothelial dysfunction, which is a prerequisite for atherosclerosis, further exacerbating structural changes and functional damage to the blood vessels. Previous studies have shown that BMI is not only an effective predictor of obesity-related diseases but also serves as a key biomarker for cardiovascular risk management in T2DM patients (57). Lifestyle modifications, particularly interventions in diet and exercise, have been shown to effectively reduce BMI levels, which multiple studies have confirmed can decrease the incidence of macrovascular complications. For example, moderate aerobic exercise and a low-calorie diet can promote fat metabolism, improve insulin sensitivity, and significantly lower blood pressure and lipid levels, thus playing an active role in reducing the occurrence of atherosclerosis and other cardiovascular events. Therefore, BMI, as a comprehensive health indicator, plays a crucial role in the management and prevention of macrovascular complications in T2DM (58–60).

Body temperature, as an emerging biomarker, is gradually gaining attention in type 2 diabetes (T2DM) patients, particularly in predicting macrovascular complications. Elevated body temperature may reflect underlying inflammation, which is closely associated with the occurrence of cardiovascular diseases, including atherosclerosis. Studies have shown that chronic low-grade inflammation typically presents as an increase in body temperature, playing a significant role in the development of atherosclerosis and other cardiovascular diseases (61). For T2DM patients, an elevated body temperature may indicate the presence of a potential inflammatory state, which accelerates vascular damage and increases the risk of heart disease, stroke, and peripheral artery disease. The relationship between body temperature and vascular complications has been well documented in the literature, with research suggesting that systemic inflammation leads to endothelial dysfunction and the formation of vascular plaques (62). Therefore, monitoring body temperature can provide valuable insights into the inflammatory processes driving macrovascular diseases in T2DM patients, potentially allowing for earlier intervention and better disease management. This finding highlights the potential clinical value of body temperature as an inflammation biomarker, particularly in the early identification and management of macrovascular complications. Additionally, elevated body temperature may influence other physiological indicators, such as respiratory rate (RR) and heart rate (HR), which may concurrently change with the body’s inflammatory response. For instance, studies have found that changes in body temperature may precede changes in heart rate and respiratory rate, suggesting that body temperature could serve as an early warning signal of disease progression (63).

Exercise frequency (EFW) is a key component of lifestyle interventions and is widely recognized for its role in cardiovascular disease prevention. Regular physical activity has been shown to significantly reduce cardiovascular disease risk by improving insulin sensitivity, lowering blood pressure, and promoting weight loss (64). Specifically, patients who engage in moderate to vigorous exercise several times a week tend to have better vascular health, with lower incidences of heart disease and stroke (65). Studies have demonstrated that increased EFW helps improve blood glucose and lipid levels, both of which are closely linked to vascular health. Additionally, sustained physical activity reduces inflammation and improves endothelial function, further lowering the risk of macrovascular complications (66). n our study, EFW was identified as an important factor influencing prediction performance in multiple machine learning models. Particularly, in the XGBoost model, EFW significantly enhanced the model’s accuracy in predicting macrovascular complications. This factor plays a critical role in the prevention and management of T2DM-related cardiovascular events (67).

Machine learning models have identified diastolic blood pressure (DBP) as a significant predictor of macrovascular complications in type 2 diabetes mellitus (T2DM) patients. Elevated DBP, in conjunction with systolic blood pressure (SBP), significantly increases the risk of cardiovascular events such as heart disease and stroke. High DBP reflects increased vascular resistance, leading to arterial wall thickening and stiffening, a recognized risk factor for cardiovascular disease in diabetic patients. Studies have shown that managing DBP is crucial for preventing the progression of macrovascular complications, as both high DBP and SBP independently contribute to the development of atherosclerosis (68, 69). Additionally, pulse pressure (PP) and the SBP/DBP ratio are significant risk factors for cardiovascular and cerebrovascular complications in diabetic patients. Research indicates that increased PP or a higher SBP/DBP ratio is strongly associated with the incidence of diabetic cardiovascular complications. Therefore, considering SBP, DBP, PP, and the SBP/DBP ratio together can provide a comprehensive assessment of cardiovascular risk in diabetic patients (70). Strict control of DBP has been shown to reduce the occurrence of cardiovascular events in T2DM patients. Expert consensus suggests that controlling the blood pressure of diabetic patients with hypertension to below 140/90 mmHg effectively reduces the risk of both macrovascular and microvascular complications (71, 72). Therefore, clinical focus should be placed on DBP management, incorporating SBP, PP, and the SBP/DBP ratio, to develop individualized treatment plans aimed at reducing cardiovascular risk in T2DM patients.

For healthcare professionals, the core of management must shift from a sole focus on glycemic control to the collaborative intervention of central obesity, chronic inflammation, and metabolic disorders. In clinical practice, it is essential to systematically assess and manage BMI, blood lipids, and blood pressure, while promoting physical activity frequency and correcting health awareness biases as first-line intervention strategies. For patients, weight loss, especially the reduction of abdominal fat, is key to preventing cardiovascular and cerebrovascular diseases. Please consider regular exercise as a core treatment, actively understand the importance of achieving comprehensive targets for blood glucose, blood lipids, and blood pressure, and actively participate in a lifestyle intervention-based comprehensive management plan.

This study still has several limitations. As a retrospective analysis relying on electronic health records, measurement errors are inevitable even after handling outliers. Self-reported lifestyle factors are subject to recall bias or reporting bias. The cohort was sourced from a single regional health management platform in Xinjiang, predominantly comprising registered local residents, which may introduce selection bias and limit the generalizability of our findings to other populations with different genetic backgrounds, lifestyles, or healthcare access. Future studies could validate and extend our findings by incorporating multi-center, prospectively designed population cohorts.

## Conclusion

We achieved the identification of the best predictive model by integrating eight traditional machine learning models with decision curve analysis. The included indicators encompass not only clinical examination indicators related to physiological and biochemical conditions but also lifestyle-related indicators from follow-up data. This comprehensive coverage is something not addressed in previous studies. The findings of this study underscore the critical importance of integrating both clinical and lifestyle-related factors for predicting macrovascular complications in Type 2 Diabetes Mellitus (T2DM). Our model, particularly the XGBoost algorithm, demonstrated superior predictive accuracy, highlighting its potential as a powerful tool for early identification of patients at high risk for macrovascular diseases. Key factors such as BMI, age, blood pressure, LDL-C, and body temperature were identified as significant predictors, reinforcing the importance of managing these parameters in clinical practice. The validation results further support the robustness and clinical applicability of our model, suggesting that machine learning algorithms, especially tree-based models like XGBoost, can aid in clinical decision-making and improve patient outcomes. Moving forward, further exploration into the interaction between these risk factors and the development of more personalized models is essential for enhancing the prediction and prevention of macrovascular complications in T2DM patients.

## Data Availability

The original contributions presented in the study are included in the article/**Supplementary Material**. Further inquiries can be directed to the corresponding author.
