# Supplementary material for: Exploring predictive factors of physiological, biochemical indicators, and lifestyle for macrovascular complications in type 2 diabetes: a synthesis of machine learning models
Source: Front Endocrinol (Lausanne). 2026 Feb 17;17:1696240. doi: 10.3389/fendo.2026.1696240 (PMC12955086; doi:10.3389/fendo.2026.1696240)
Supplement: Supplementary file 1 [file Table1.docx]

**Supporting Information 1. Table S1: Baseline Demographic Characteristics Based on ML Training and Validation Set Partitioning**

**Table S1: Baseline Demographic Characteristics Based on ML Training and Validation Set Partitioning**

| **Characteristics** | **Total cohort**  **(N=4186)** | **Training cohort**  **(n=3348)** | **validation cohort**  **(n=838)** | ***P-value*** |
| --- | --- | --- | --- | --- |
| **Gender(0: Male, Female: Yes)** | |  |  | 0.94 |
| Male,n/total(%) | 2285(54.5%) | 1825(54.5%) | 460(54.8%) |  |
| Female,n/total(%) | 1901(45.4%) | 1523(45.4%) | 378(45.1%) |  |
| **Age(years)** | |  |  | 0.0455 |
| <=50 | 356(8.5%) | 267(8.0%) | 89(10.6%) |  |
| <=60 | 1899(45.4%) | 1519(45.4%) | 380(45.3%) |  |
| <=70 | 1356(32.4%) | 1087(32.4%) | 269(32.1%) |  |
| >70 | 575(13.7%) | 475(14.2%) | 100(11.9%) |  |
| **Ethnicity(categorical)** | |  |  | 0.63 |
| Uighur nationality | 4128(98.6%) | 3300(98.5%) | 828(98.8%) |  |
| Other | 58(1.3%) | 48(1.4%) | 10(1.1%) |  |
| **Marital** |  |  |  | <0.001 |
| Married | 3747(89.2%) | 2992(89.3%) | 745(88.9%) |  |
| Single | 449(10.7%) | 357(10.6%) | 93(10.0%) |  |
| **Number of cases(%)** | 481(11.5%) | 385(11.5%) | 96(11.4%) | 0.84 |
| Male | 265(11.5%) | 212 (11.5%) | 53 (11.4%) |  |
| Female | 216(11.3%) | 173 (11.3%) | 43 (11.3%) |  |

Note:Daily Smoking Consumption (cigarettes/day), Daily Drinking Consumption (units/day), Exercise Frequency per week (times/week), and Exercise Duration (minutes).
