# Supplementary material for: Exploring predictive factors of physiological, biochemical indicators, and lifestyle for macrovascular complications in type 2 diabetes: a synthesis of machine learning models
Source: Front Endocrinol (Lausanne). 2026 Feb 17;17:1696240. doi: 10.3389/fendo.2026.1696240 (PMC12955086; doi:10.3389/fendo.2026.1696240)
Supplement: Supplementary file 2 [file Table2.docx]

**Supporting Information 2. Table S2: Clinical and Biochemical Characteristics Based on ML Training and Validation Set Partitioning**

**Table S2: Clinical and Biochemical Characteristics Based on ML Training and Validation Set Partitioning**

| **Characteristics** | **Total cohort**  **(N=4186)** | **Training cohort**  **(n=3348)** | **Validation cohort(n=838)** | ***P-value*** |
| --- | --- | --- | --- | --- |
| Lower Extremity Edema  (0: No, 1: Yes) | 26(0.6%) | 21(0.6) | 5(0.6%) | 0.96 |
| Hypoglycemic Reaction  (0: No, 1: Yes) | 83(1.9%) | 70(2.1%) | 13(1.6%) | 0.53 |
| Body Temperature(°C)(mean(SD)) | 36.34(0.193) | 36.34 (0.1946) | 36.33 (0.1857) | 0.91 |
| Body Mass Index(kg/m²) | 26.53(3.35) | 26.47 (3.34) | 26.76 (3.38) | 0.31 |
| Diastolic Blood Pressure(mmHg) | 76.6(13.65) | 76.50 (11.92) | 76.99 (19.06) | 0.71 |
| Systolic Blood Pressure(mmHg) | 129.3(13.78) | 129.30 (13.81) | 129.48 (13.66) | 0.85 |
| Hearing Ability  (0: Normal, 1: Impaired) | 63(1.5%) | 51(1.5%) | 12(1.4%) | 0.97 |
| Respiratory Rate(breaths per minute) | 78.8(11.13) | 20.06 (3.36) | 19.97 (2.27) | 0.55 |
| Pharyngeal Throat  (0: Normal, 1: Abnormal) | 13(0.3%) | 11(0.3%) | 2(0.2%) | 0.72 |
| Scleral Check  (0: Normal, 1: Abnormal) | 17(0.4%) | 15(0.4%) | 2(0.2%) | 0.44 |
| Heart Rate(beats per minute) | 78.8(11.1) | 78.78 (11.20) | 78.83 (10.91) | 0.99 |
| Total Cholesterol(mmol/L) | 4.8(7.99) | 4.85 (8.91) | 4.64 (1.37) | 0.23 |
| Total Bilirubin(µmol/L) | 12.9(7.86) | 12.92 (7.49) | 13.12 (9.23) | 0.78 |
| Triglycerides(mmol/L) | 2.08(1.94) | 2.06 (1.92) | 2.14 (2.03) | 0.49 |
| White Blood Cell(10^9/L) | 7.37(8.63) | 7.39 (9.38) | 7.27 (4.56) | 0.95 |
| Albumin(g/L) | 44.0(10.44) | 44.02 (7.16) | 44.01 (18.44) | 0.99 |
| Fasting Blood Glucose(mmol/L) | 9.24 (5.05) | 9.25 (5.07) | 9.22 (5.00) | 0.92 |
| Direct Bilirubin(µmol/L) | 4.65 (5.59) | 4.70 (5.94) | 4.45 (3.93) | 0.56 |
| Hepatomegaly(0: No, 1: Yes) | 8(0.2%) | 6(0.2%) | 2(0.2%) | 0.81 |
| Lung Breath Sounds  (0: Normal, 1: Abnormal) | 30(0.7%) | 24(0.7%) | 6(0.7%) | 0.96 |
| Rales(0: No, 1: Yes) | 26(0.6%) | 19(0.6%) | 7(0.8%) | 0.71 |
| Pulse Rate(beats per minute) | 80.04 (11.16) | 80.00 (11.19) | 80.22 (11.04) | 0.93 |
| Splenomegaly(0: No, 1: Yes) | 7(0.2%) | 5(0.1%) | 2(0.2%) | 0.81 |
| Waist Circumference(cm) | 92.94(12.10) | 92.79 (12.21) | 93.54 (11.67) | 0.32 |
| Abdominal Mass(0: No, 1: Yes) | 11(0.3%) | 8(0.2%) | 3(0.4%) | 0.64 |
| Abdominal Tenderness  (0: No, 1: Yes) | 31(0.7%) | 24(0.7%) | 7(0.8%) | 0.91 |
| Platelets(10^9/L) | 249.78(70.30) | 249.09 (70.69) | 252.56 (68.69) | 0.47 |
| Blood Urea(mmol/L) | 5.23 (2.92) | 5.25 (2.95) | 5.12 (2.80) | 0.51 |
| Low-Density Lipoprotein Cholesterol  (mmol/L) | 2.70 (1.09) | 2.70 (1.11) | 2.69 (1.05) | 0.91 |
| Serum Creatinine(µmol/L) | 61.46 (37.77) | 61.74 (40.53) | 60.34 (23.69) | 0.78 |
| Alanine Aminotransferase(U/L) | 32.96(280.41) | 30.86 (224.93) | 41.35 (436.75) | 0.56 |
| Aspartate Aminotransferase(U/L) | 23.51 (33.75) | 23.61 (37.13) | 23.14 (13.50) | 0.93 |
| Hemoglobin(g/L) | 145.74(21.01) | 145.42 (21.23) | 147.02 (20.07) | 0.57 |
| Height(cm) | 160.57 (8.28) | 160.54 (8.21) | 160.69 (8.56) | 0.81 |
| Barrel Chest(0: No, 1: Yes) | 14(0.3%) | 9(0.3%) | 5(0.6%) | 0.61 |
