# Supplementary material for: Exploring predictive factors of physiological, biochemical indicators, and lifestyle for macrovascular complications in type 2 diabetes: a synthesis of machine learning models
Source: Front Endocrinol (Lausanne). 2026 Feb 17;17:1696240. doi: 10.3389/fendo.2026.1696240 (PMC12955086; doi:10.3389/fendo.2026.1696240)
Supplement: Supplementary file 3 [file Table3.docx]

**Supporting Information 3. Table S3: Baseline Lifestyle Characteristics Based on ML Training and Validation Set Partitioning**

**Table S3: Baseline Lifestyle Characteristics Based on ML Training and Validation Set Partitioning**

| **Characteristics** | **Total cohort**  **(N=4186)** | **Training cohort**  **(n=3348)** | **Validation cohort**  **(n=838)** | ***P-value*** |
| --- | --- | --- | --- | --- |
| Smoking Status(0: Non-smoker, 1: Smoker) | 249(5.9%) | 197(5.9%) | 52(6.2%) | 0.89 |
| Years of Exercise (years) | 0.17 (1.37) | 0.18 (1.46) | 0.16 (0.94) | 0.79 |
| Daily Smoking Consumption (cigarettes/day) | 0.74 (3.83) | 0.73 (3.83) | 0.75 (3.83) | 0.94 |
| Daily Drinking Consumption (units/day) | 0.62(12.60) | 0.54(11.87) | 0.93 (15.14) | 0.08 |
| Per Exercise Time (minutes) | 1.31 (7.29) | 1.23 (6.96) | 1.62 (8.48) | 0.56 |
| Psychological Adjustment (0: Poor, 1: Good) | 34(0.8%) | 30(0.9%) | 4(0.5%) | 0.54 |
| Physical Functional Status(0: Poor, 1: Good) | 35(0.8%) | 27(0.8%) | 2(0.7%) | 0.79 |
| Exercise Duration(minutes) | 11.92(15.51) | 11.99 (15.97) | 11.67 (13.49) | 0.96 |
| Exercise Frequency per week(times/week) | 2.03 (2.54) | 2.03 (2.56) | 2.01 (2.46) | 0.85 |
| Genetic History(0: No, 1: Yes) | 183(4.4%) | 142(4.2%) | 41(4.9%) | 0.46 |
| Exercise Frequency (times/week) | 1.13 (0.57) | 1.13 (0.57) | 1.14 (0.60) | 0.71 |
| Drinking Frequency (0: No, 1: Yes) | 112(2.7%) | 86(2.6%) | 26(3.1%) | 0.62 |
